# Supplementary material for: GFAPβ and GFAPδ Isoforms Expression in Mesenchymal Stem Cells, MSCs Differentiated Towards Schwann-like, and Olfactory Ensheathing Cells
Source: Curr Issues Mol Biol. 2025 Jan 9;47(1):35. doi: 10.3390/cimb47010035 (PMC11764465; doi:10.3390/cimb47010035)
Supplement: Supplementary file 1 [file cimb-47-00035-s001.zip › cimb-3386153-supplementary.pdf]

Supplementary Material

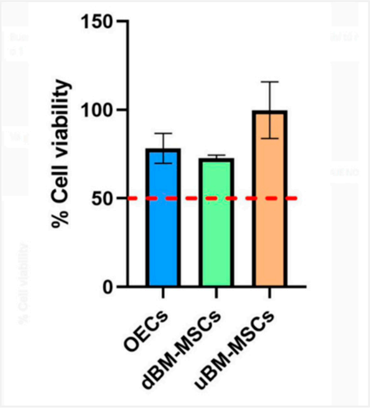

Supplementary material Figure S1. Cell viability by MTT assay.

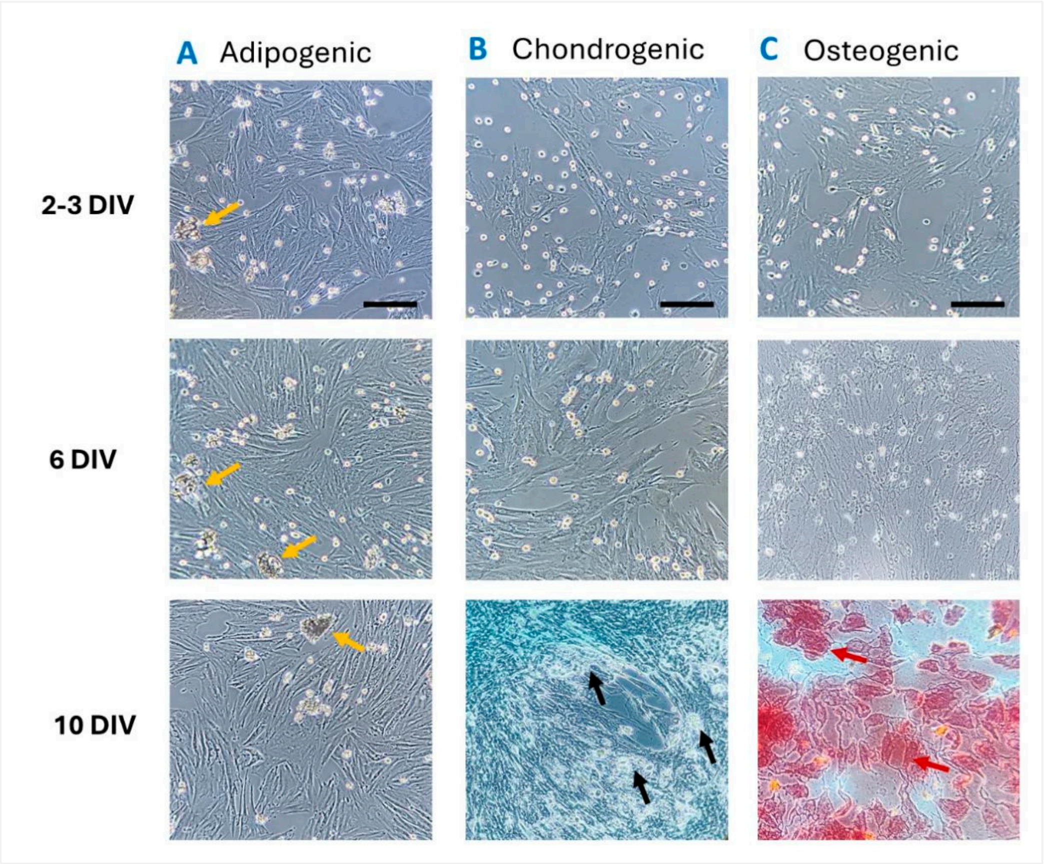

Supplementary material Figure S2. BMSCs differentiation was identify by morphology of (A) adipogenic, (B) chondrogenic and (C) osteogenic differentiation potential. Bar 100 µm.

Supplementary material data analysis

GFAPα

Descriptive values of GFAPα	OECs	dBM-MSCs	uBM-MSCs
Number of values	3	3	3
Minimum	7.125	2.460	1.760
Maximum	15.58	6.323	3.289
Range	8.455	3.863	1.529
Mean	10.96	3.993	2.479
Std. Deviation	4.282	2.051	0.7686
Std. Error of Mean	2.472	1.184	0.4437

ANOVA summary of GFAPα	
F	7.955
P value	**0.0205**
P value summary	*
Significant diff. among means (P < 0.05)?	Yes
R square	0.7261

ANOVA table of GFAPα	SS	DF	MS	F (DFn, DFd)	P value
Treatment (between columns)	122.7	2	61.35	F (2, 6) = 7.955	**P=0.0205**
Residual (within columns)	46.27	6	7.712		
Total	169.0	8			

Tukey's multiple comparisons test of GFAPα	Mean Diff.	95.00% CI of diff.	Adjusted P Value
OECs vs. dBM-MSCs	6.965	0.007562 to 13.92	**0.0498**
OECs vs. uBM-MSCs	8.479	1.522 to 15.44	**0.0225**
dBM-MSCs vs. uBM-MSCs	1.514	-5.443 to 8.471	0.7897

GFAPβ

Descriptive values of GFAPβ	OECs	dBM-MSCs	uBM-MSCs
Number of values	3	3	3

Minimum	8.265	0.7130	0.1290
Maximum	10.71	1.233	0.5620
Range	2.447	0.5200	0.4330
Mean	9.826	1.033	0.3423
Std. Deviation	1.356	0.2798	0.2166
Std. Error of Mean	0.7830	0.1615	0.1250

**ANOVA summary of GFAPβ**	
F	128.1
P value	**<0.0001**
P value summary	****
Significant diff. among means (P < 0.05)?	Yes
R square	0.9771

**ANOVA table of GFAPβ**	SS	DF	MS	F (DFn, DFd)	P value
Treatment (between columns)	167.8	2	83.88	F (2, 6) = 128.1	**P<0.0001**
Residual (within columns)	3.929	6	0.6548		
Total	171.7	8			

**Tukey's multiple comparisons test of GFAPβ**	Mean Diff.	95.00% CI of diff.	Adjusted P Value
OECs vs. dBM-MSCs	8.794	6.766 to 10.82	**<0.0001**
OECs vs. uBM-MSCs	9.484	7.457 to 11.51	**<0.0001**
dBM-MSCs vs. uBM-MSCs	0.6903	-1.337 to 2.718	0.5785

GFAPδ

**Descriptive values of GFAPδ**	OECs	dBM-MSCs	uBM-MSCs
Number of values	3	3	3
Minimum	4.190	4.540	3.151
Maximum	10.93	12.92	10.50
Range	6.739	8.382	7.344
Mean	7.603	8.490	7.545
Std. Deviation	3.370	4.212	3.879
Std. Error of Mean	1.946	2.432	2.240

**ANOVA summary of GFAPδ**	
F	0.05719
P value	0.9449
P value summary	ns
Significant diff. among means (P < 0.05)?	No
R square	0.01871
